# Anion‐Specific Mechanisms in Fibrinogen Self‐Assembly: Contrasting Effects of Phosphates and Chlorides in Nanofiber Formation

**DOI:** 10.1002/mabi.70203

**Published:** 2026-06-14

**Authors:** Antoine Eyram Kwame, Aparna Sai Malisetty, Michael Maas, Susan Köppen‐Hannemann, Lucio Colombi Ciacchi, Dorothea Brüggemann

**Affiliations:** ^1^ Biophysics and Applied Biomaterials Hochschule Bremen – City University of Applied Sciences Bremen Germany; ^2^ Hybrid Materials Interfaces Group Faculty of Production Engineering and Bremen Center For Computational Materials Science University of Bremen Bremen Germany; ^3^ Advanced Ceramics University of Bremen Bremen Germany; ^4^ MAPEX Center for Materials and Processes University of Bremen Bremen Germany

**Keywords:** anion‐induced protein aggregation, environmental scanning electron microscopy, fibrinogen nanofibers, ion‐protein interactions, light scattering techniques, tissue engineering scaffolds

## Abstract

Fibrinogen can self‐assemble into nanofibers in the presence of salts without thrombin. Although kosmotropic anion‐cation pairs are known to govern this process, the role of individual anions remains unclear. Here, we demonstrate that fibrinogen self‐assembly follows a strongly anion‐specific concentration‐dependent pathway. Moderate phosphate concentrations produced interconnected nanofibrous networks that formed stepwise, whereas higher phosphate and all chloride conditions yielded macroporous aggregates. Elemental analysis revealed retention of sodium and phosphate within the fibers, indicating specific ion–protein interactions. Light‐scattering analyses showed sigmoidal kinetics and concentration‐dependent growth with phosphate, consistent with nucleation and fibril elongation, whereas chloride produced non‐sigmoidal behavior indicative of disordered aggregation. Together, these results demonstrate that multivalent phosphate promotes controlled fibrillogenesis of fibrinogen, while monovalent chloride favors amorphous precipitation. This anion‐directed assembly pathway provides an enzyme‐free route to fabricate fibrinogen nanofibers with tunable architecture for various biomaterial applications.

## Graphical abstract

Fibrinogen self‐assembly follows distinct anion‐directed pathways. Phosphate promotes ordered nucleation and elongation into interconnected nanofibrous networks, whereas chloride induces disordered porous aggregates. Specific phosphate retention within the fibers suggests direct ion–protein interactions during fibrillogenesis. The findings provide an enzyme‐free strategy to tailor fibrinogen nanostructures and architectures for biomaterial applications.

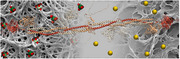

## 1 Introduction

Fibrinogen is a large, complex glycoprotein with a molecular weight of approximately 340 kDa [1, 2, 3], consisting of three pairs of polypeptide chains (Aα, Bβ, and γ) linked by 29 disulfide bonds. This unique architecture is fundamental to its biological function and self‐assembly properties. Under physiological conditions, fibrinogen serves as the precursor to fibrin, which results from thrombin‐mediated cleavage of fibrinopeptides A and B from the fibrinogen molecule. This step exposes polymerization sites that enable fibrin monomers to assemble into protofibrils [2], which subsequently aggregate to form a three‐dimensional fibrin network essential for hemostasis [4]. The resulting fibrin nanofibers are insoluble and provide a scaffold for platelet adhesion, which helps to prevent blood loss during injury [2], and also functions as a provisional extracellular matrix during tissue repair [5].

Many in vitro strategies have been developed to replicate this 3D nanostructure from fibrinogen, including different self‐assembly methods based on substrate interactions or denaturing and non‐denaturing buffer systems [6]. Electrospinning, which also uses denaturing buffer conditions, can produce dense fibrillar networks with excellent biocompatibility [7, 8, 9, 10]. However, it is not a resource‐efficient approach to prepare fibrinogen nanofibers for tissue engineering because it requires high protein concentrations and the use of high voltage, which can denature the protein [11, 12]. These limitations might be overcome by the salt‐induced self‐assembly method [13, 14], which creates a high‐salinity microenvironment during evaporation of a fibrinogen solution, facilitating protein aggregation into nanofibrous networks. This approach avoids protein denaturation [13], and the resulting fibers closely resemble native fibrin networks in morphology and mechanical properties [15, 16]. With these features, fibrinogen nanofibers prepared by salt‐induced self‐assembly have already been shown to support platelet adhesion [17], and the co‐cultivation of fibroblasts and keratinocytes [18].

Although Ca^2+^ ions play a critical role during fibrin polymerization in vivo [2], divalent cations like Mg^2+^, Ca^2+^, Zn^2+,^ or Cu^2+^ were not found to induce fiber assembly of fibrinogen [19]. Instead, fibrinogen fibers exclusively formed in the presence of monovalent cations, and we recently attributed this effect to cooperative kosmotropic anion‐cation effects [20]. Interestingly, Hense and Strube recently showed that Mg^2+^ and Ca^2+^ ions could induce gelation of fibrinogen into fibrous hydrogels in the absence of thrombin when low temperatures of 5°C and long incubation times up to 24 h were used, and at physiological conditions, respectively [21, 22]. Also, with low concentrations of sodium phosphate and sodium citrate, fibrinogen could spontaneously organize into nanofibers in solution, a process that speeds up when the ionic strength is reduced [23]. It was concluded that suitable triggers for fibrillogenesis in the presence of monovalent Na^+^ cations are oxygen‐containing, preferably multivalent acid anions [24]. This assumption was recently confirmed in our work, where fibrinogen assembled into fibers upon drying with sodium sulfate [20].

Another study by Strube and co‐workers focused on the role of monovalent cations during fiber assembly of fibrinogen. Using a mixture of sodium chloride and sodium phosphate, they demonstrated that a reduction in ionic strength reduced electrostatic screening and triggered a stepwise coalescence process [23]. Fibrinogen first formed globular clusters, which then merged and reorganized into fibril‐like, interconnected networks [25]. Interestingly, this observation contradicts our recent finding that high initial salt concentrations up to 150 mM Na‐PO_4_ could promote fibrillogenesis [20]. When we exemplarily considered the individual role of sodium phosphate and sodium chloride during fiber assembly, fiber formation was more pronounced for phosphate than for chloride anions, which was found to correlate with a much tighter and more persistent adsorption of phosphate anions in molecular dynamics simulations [20]. At the same time, we observed significant differences in cation interaction with the fibrinogen molecule in dependence on the anion. Only when fibrinogen fibers were formed with phosphate anions, a retention of Na^+^ ions was observed following washing, which did not occur with chloride anions [20].

Despite these previous studies, there is still a lack of systematic understanding of how chloride and phosphate anions steer fibrinogen fiber assembly in dependence on the salt and protein concentration and how cation interactions with fibrinogen are modulated by these anions. In particular, the kinetics of fiber assembly are still not fully understood. Unravelling these mechanisms is crucial to provide a general concept for the fiber assembly of fibrinogen in a thrombin‐free environment and to develop clinically relevant fibrinogen‐based biomaterials. Therefore, we studied the concentration‐dependent effects of chloride and phosphate anions on fibrinogen assembly upon drying, hypothesizing that chaotropic chloride induces disordered aggregation while phosphate favors ordered nanofibril formation through kosmotropic and ion‐specific interactions. Unlike previous studies that employ mixed salt systems [13, 14, 25], or treat ionic strength as the primary variable [23], this present work systematically disentangles the individual contributions of phosphate and chloride anions across a range of both protein and salt concentrations. This enables, for the first time, a mechanistic framework for anion‐directed, enzyme‐free fibrinogen fibrillogenesis independent of thrombin.

## 2 Materials and Methods

### 2.1 Buffers and Salt Solutions

All buffers and salt solutions were prepared using deionized water from a TKA water purification system (Thermo Fisher Scientific, Schwerte, Germany). The pH was monitored and adjusted with a pH meter (pH 50 Violab, Italy) by dropwise addition of concentrated HCl or NaOH solution (both VWR, Darmstadt, Germany). (Tris(hydroxymethyl)‐aminomethane buffer solutions of (C_4_H_11_NO_3_, Tris Carl Roth, Karlsruhe, Germany) were prepared with a concentration of 10 mM and a pH of 7.0. Phosphate‐buffered saline (PBS, Gibco, Thermo Fisher) stock solutions were prepared at concentrations of 5× and 15× with a pH of 7.4. Sodium phosphate (Na‐PO_4_) buffer with stock concentrations of 300 and 2250 mM was prepared by mixing NaH_2_PO_4_ and Na_2_HPO_4_ (Carl Roth) solutions in varying ratios to achieve pH 7.4. The same concentrations and pH were used for potassium phosphate (K‐PO_4_) by mixing KH_2_PO_4_ and K_2_HPO_4_ (Sigma–Aldrich, Steinheim, Germany). Stock solutions of sodium chloride (NaCl, VWR) and potassium chloride (KCl, Carl Roth) were also prepared at 300 and 2250 mM with pH 7.4.

### 2.2 Fibrinogen Solutions and Self‐Assembly

Fibrinogen was a research material derived from human blood plasma provided by Biotest AG (Biotest, Dreieich, Germany). Fibrinogen was dissolved in sterile water, followed by overnight dialysis in 10 mM Tris using 14 kDa cutoff cellulose membrane dialysis tubing (Sigma) to remove low molecular weight compounds. Prior to fiber assembly, round glass slides (VWR) with a diameter of 15 mm were cleaned in an air plasma for 2 min using a plasma cleaner (Diener electronic GmbH, Ebhausen, Germany).

Fibrinogen nanofibers were prepared by pipetting 100 µL of the fibrinogen stock solution and 100 µL of the respective salt solution onto the plasma‐cleaned glasses. All samples were placed in a humidity chamber (Memmert, Schwabach, Germany) and dried at 25°C and 30% relative humidity overnight. The samples were subsequently crosslinked for 2 h by placing them in a petri dish containing one microliter of 37% formaldehyde solution (FA, AppliChem GmbH, Germany) per cm^3^, covered with parafilm. Afterward, the samples were aired in the fume hood for 30 min and rinsed with 1 mL deionized water for 15 min (exchanging the water every 5 min).

### 2.3 Dynamic Light Scattering Measurements

Dynamic light scattering (DLS) was employed to measure the hydrodynamic radius (R_h_) distribution of fibrinogen molecules in solution under various ionic conditions. Fibrinogen samples were prepared at a concentration of 0.5 mg/mL in Tris‐HCl buffer containing 50 mM, 100 mM, 150 mM, 300 mM, 600 mM, and 1 M of sodium phosphate and sodium chloride, respectively. Measurements were conducted using a Zetasizer Nano ZSP (Malvern Panalytical, Worcestershire, United Kingdom) equipped with a 633 nm He–Ne laser at a scattering angle of 173° (backscatter configuration). Samples were equilibrated at 25°C for 3 min before data acquisition. Each measurement consisted of 10 to 15 runs and was measured in triplicate to ensure reproducibility. The intensity‐weighted size distribution was analyzed using the instrument's software, applying cumulant analysis to determine the hydrodynamic radius. Results were reported as mean ± standard deviation from at least three independent measurements. The hydrodynamic radius R_h_ was obtained via the Stokes−Einstein relation:

$$\;R_{h}=\frac{k_{B}T}{6\pi \eta D}$$

where k_B_ is the Boltzmann constant, T is the absolute temperature, and η = 0.89 cP is the dynamic viscosity of water at 25°C. All measurements were performed at t = 0 h and t = 3 h, respectively.

### 2.4 Turbidity Measurements and Mass Loss Analysis

To follow the nanofiber formation process upon the addition of different salts, the absorbance at 330 nm [26] was continuously recorded for 72 h at 25°C using a Multiskan Sky microplate spectrophotometer (Thermo Fisher). The measurements were conducted in a Greiner 96‐well plate (Sigma–Aldrich) containing samples of 50 µL fibrinogen at final concentrations of 2.5, 5, 10, and 15 mg/mL, prepared in Tris buffer with 50 µL of the respective salt solutions at varying concentrations. The turbidity‐time graphs of fibrinogen in the different salt solutions provided information on the different phases/stages of the nanofiber formation. The slopes of the turbidity‐time curve provide insight into the rate of fiber assembly and salt crystallization.

To monitor solvent evaporation and fibrinogen retention during drying, we conducted simultaneous mass‐loss experiments following our previous routine [20]. Fibrinogen samples were loaded into well plates and placed in a UV/vis spectrophotometer, where turbidity (absorbance at 330 nm) was recorded at hourly intervals without disturbing the sample. Immediately after each absorbance measurement, the well‐plate was removed, briefly covered to minimize moisture uptake, and weighed using an analytical balance (Kern PCB, Regensburg, Germany) to determine the remaining mass of the fibrinogen‐salt solution. The initial sample mass was recorded before drying commenced. At each time point, the decrease in mass was attributed to solvent (water) evaporation, allowing calculation of the residual salt mass in the fibrinogen precipitate. This procedure was repeated until the mass plateaued, indicating complete drying. These experiments were performed at room temperature under ambient humidity conditions.

### 2.5 Light and Scanning Electron Microscopy

The surface coverage and microscopic features of dried fibrinogen precipitates before cross‐linking and washing were imaged using a Keyence VHX‐7000 digital microscope (Keyence, Neu‐Isenburg, Germany). Images were captured at 20× magnification for full‐sample surveys through stitching.

Subsequently, the nanoscale morphology of the fibrinogen precipitates was examined with scanning electron microscopy (SEM). Unless stated otherwise, prior to imaging, the samples were sputter‐coated with a 7 nm gold layer using an EM ACE600 high‐vacuum sputter coater (Leica Microsystems, Wetzlar, Germany). SEM imaging was performed with a Quattro S (ThermoFisher) at an acceleration voltage of 5 kV using a secondary electron detector. Fiber diameters and pore sizes were quantified from SEM images using ImageJ/FIJI and the BoneJ plugin [27].

To follow morphological changes during the fiber formation, we performed SEM analysis of uncoated fibrinogen precipitates in low‐vacuum mode. For this purpose, fibrinogen‐salt solutions were pipetted onto clean glass coverslips and incubated for different time intervals (15, 30, 45, and 60 min) under ambient conditions. The remaining protein solution was carefully removed at each time point and allowed to dry through slow evaporation. The dried precipitates were directly imaged in low‐vacuum mode (70 Pa) without sputtering at an acceleration voltage of 5 kV. Afterward, they were sputter‐coated with gold to perform a complementary SEM analysis in high‐vacuum mode.

### 2.6 EDS Analysis of Fibrinogen Nanofibers or Precipitates

Energy‐dispersive X‐ray spectroscopy (EDS) was used to analyze the elemental composition of dried fibrinogen precipitates containing different salts. The samples were not sputter‐coated and were imaged in low vacuum mode (75 Pa). EDS measurements were taken with the EDS detector of the Quattro S system. The accelerating voltage was set to 10 kV, and the working distance was maintained at 10 mm. Spectra were collected from selected areas of interest through mapping, and elemental quantification was performed.

## 3 Results and Discussion

### 3.1 Morphology of Surfaces Formed With Different Salts

**FIGURE 1:**
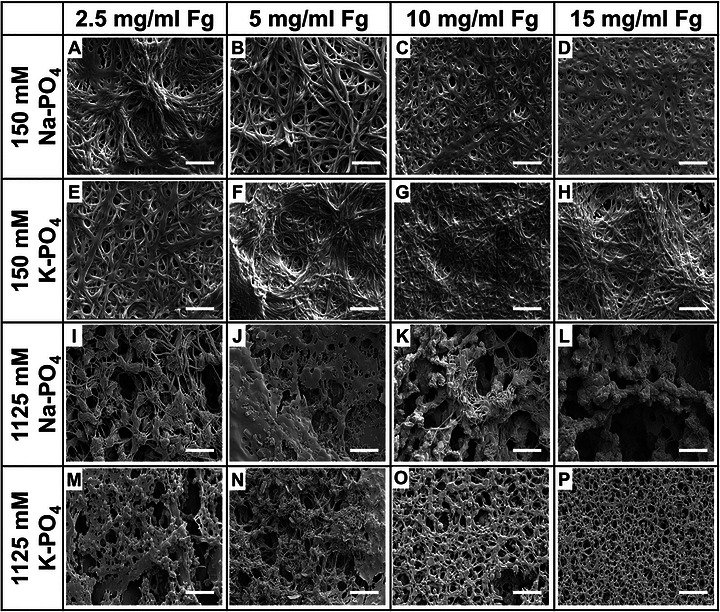
SEM images of the morphology of fibrinogen precipitates, which were assembled with different fibrinogen starting concentrations in the presence of two different starting concentrations of phosphate‐containing salts, subsequently dried, crosslinked with FA vapor, and washed. When fibrinogen from (A) 2.5 mg/mL, (B) 5 mg/mL, (C) 10 mg/mL, and (D) 15 mg/mL solutions was dried in the presence of 150 mM Na‐PO_4_, fiber networks with varying diameters and pore sizes were found. The networks were less porous for higher protein concentrations. Fibrinogen from (E) 2.5 mg/mL to (F) 5 mg/mL, (G) 10 mg/mL, and (H) 15 mg/mL solutions with 150 mM K‐PO_4_ also yielded nanofiber networks, which appeared denser for higher fibrinogen concentrations. When (I) 2.5 mg/mL to (J) 5 mg/mL, (K) 10 mg/mL, and (L) 15 mg/mL fibrinogen was dried with 1125 mM Na‐PO_4_, and (M) 2.5 mg/mL to (N) 5 mg/mL, (O) 10 mg/mL and (P) 15 mg/mL fibrinogen was dried in the presence of 1125 mM K‐PO_4_ the morphology varied from that at lower salt concentration, and porous, spongy precipitates were found that did not exhibit defined fiber. Overall, 150 mM of both phosphates favored the formation of well‐defined and porous fibrinogen nanofibers, while 1125 mM tended to suppress fiber assembly. Scale bar represents 5 µm.

When fibrinogen at different starting concentrations was dried with sodium or potassium phosphate, SEM analysis showed clear morphological differences (see Figure 1) that correlated with the macroscopic appearance of the precipitates (see Figures S1 and S2). Phosphate anions consistently promoted nanofiber formation, but the overall network structure strongly depended on the protein concentration. At very low fibrinogen concentrations, evaporation‐driven assembly does not yield reproducible fibrous networks due to insufficient protein mass to sustain network formation during drying, consistent with prior work [13, 14].

With 150 mM sodium phosphate, 2.5 mg/mL fibrinogen formed nanofibers (187 ± 41.7 nm) with small pores (137 ± 31.4 nm), as shown in Figures 1A and S3. At 5 mg/mL, (Figure 1B) significantly thicker fibers (226.3 ± 47.6 nm) and larger pores (216 ± 58.1 nm) produced a highly porous network. In contrast, higher fibrinogen concentrations of 10 and 15 mg/mL yielded denser, less porous networks (Figure 1C,D) with smaller fiber diameters (138 ± 25.9 and 123.7 ± 19.5 nm) and reduced pore sizes (82.9 ± 19.2 and 80.3 ± 23.4 nm). All precipitates appeared smooth and free of visible salt crystals (see Figure S1A–D), and the range of fiber diameters agrees well with our previous study where phosphate‐buffered saline (PBS) was used to induce fiber assembly [13, 16].

To assess potential cationic effects on fiber assembly, fibrinogen was also dried with 150 mM potassium phosphate (see Figure 1E–H). Compared to sodium phosphate, precipitates appeared less uniform and displayed ring‐like patterns (see Figure S1E–H). At 2.5 mg/mL, nanofibers showed thicker diameters and larger pores than with sodium phosphate, while at 5 to 15 mg/mL, nanofibers formed compact, bundled networks with minimal porosity, resembling dense structures observed at high fibrinogen concentrations with sodium phosphate. This concentration‐dependent compaction is consistent with macromolecular crowding effects, wherein excluded volume interactions can accelerate nucleation kinetics and promote lateral fiber association over longitudinal elongation, resulting in denser networks with reduced pore size, as reported for other filamentous protein systems [28, 29, 30]. This interpretation is further supported by established excluded volume theories of macromolecular crowding [31, 32, 33]. Thus, while 150 mM potassium phosphate supported fibrillogenesis, it produced more compact and less porous networks, making it less suitable for tissue engineering applications that require cell migration and nutrient transfer [34].

Increasing the sodium phosphate concentration to 1125 mM progressively disrupted fibrillogenesis (Figure 1I–L) for all fibrinogen concentrations. At 2.5 mg/mL, only partial nanofibrous structures with thin nanofibers and large voids were observed, while for 5 to 15 mg/mL, the morphology shifted from irregular aggregates with few nanofibers to macroporous or dense amorphous clumps lacking defined nanofibers. Similar trends were observed with 1125 mM potassium phosphate (Figure 1M–P), yielding heterogeneous, sponge‐like, or aggregated structures depending on the fibrinogen concentration.

The morphological differences between Na‐PO_4_ and K‐PO_4_ networks can be rationalized by the distinct hydration properties of the two cations. Na^+^ (ionic radius 0.102 nm; hydration energy ≈ −405 kJ/mol) is more strongly hydrated than K^+^ (0.138 nm; ≈ −321 kJ/mol) [19], while K^+^ ions also tend to reside further from the protein surface than Na^+^ ions [20]. Together, these differences may promote more stable ion‐pair interactions with phosphate at the protein surface. Consistent with this interpretation, previous XPS data from Stamboroski et al. [20], showed that Na^+^ is retained on fiber surfaces after washing, while K^+^ is not, suggesting that Na^+^ participates more readily in persistent phosphate‐mediated interactions with fibrinogen. This difference may contribute to the more compact morphology observed for K‐PO_4_‐induced networks.

**FIGURE 2:**
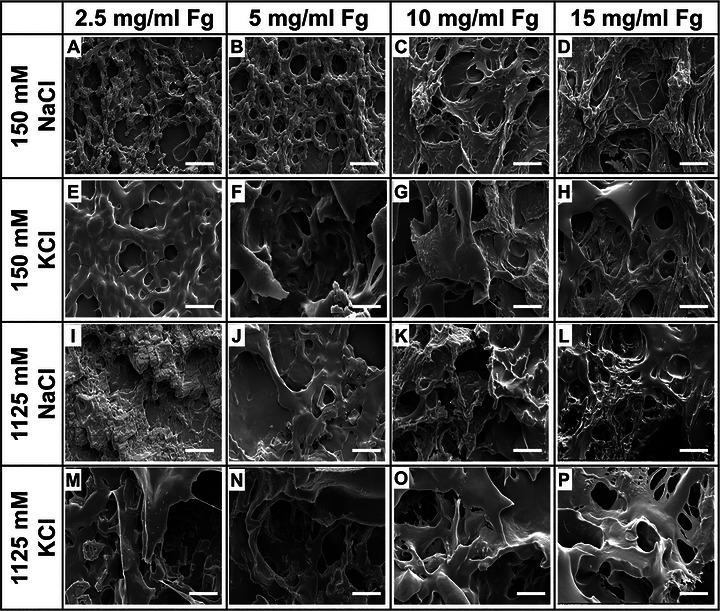
SEM images showing the morphology of fibrinogen precipitates assembled with different fibrinogen concentrations in the presence of two different chloride‐containing salts, then dried, FA vapor crosslinked, and washed. Fibrinogen from (A) 2.5 mg/mL, (B) 5 mg/mL, (C) 10 mg/mL, and (D) 15 mg/mL dried in 150 mM NaCl showed no nanofibers. Fibrinogen from (E) 2.5 mg/mL to (F) 5 mg/mL, (G) 10 mg/mL, and (H) 15 mg/mL with 150 mM KCl also yielded macroporous precipitates. When (I) 2.5 mg/mL to (J) 5 mg/mL, (K) 10 mg/mL, and (L) 15 mg/mL fibrinogen was dried with 1125 mM NaCl, and (M) 2.5 mg/mL to (N) 5 mg/mL, (O) 10 mg/mL, and (P) 15 mg/mL fibrinogen were dried with 1125 mM KCl, all showed macroporous surfaces. Scale bar represents 5 µm.

To evaluate anionic effects, fibrinogen was dried with 150 and 1125 mM NaCl or KCl, respectively. In contrast to phosphate salts, no defined fiber networks formed at any fibrinogen concentration, as seen in Figure 2A–P. Instead, nodular, rough, or molten aggregates were observed. Higher chloride concentrations yielded similarly rugged morphologies independent of cation or protein concentration. Macroscopically, chloride samples displayed visible dendritic salt crystals (see Figure S3A–P), agreeing well with our previous study on protein‐salt interactions [35]. These findings indicate that fibrinogen–anion interactions govern whether ordered nanofiber formation is promoted, as seen for phosphates or suppressed, as observed with chlorides.

When fibrinogen was dried with 2.5× and 7.5× PBS, the resulting structures showed mixed features of phosphate‐ and chloride‐containing systems, as PBS contains both anions (see Figure S4A–H). More pronounced nanofiber formation was observed with 2.5× PBS than with 7.5× PBS, supporting our hypothesis that fiber assembly is hindered above critical salt or protein concentrations. The precipitate morphology further suggests competition between fiber‐promoting phosphate ions and fiber‐inhibiting chloride ions within the same buffer.

### 3.2 Elemental Analysis of Fibrinogen Precipitates With EDS

Our previous studies showed that fibrinogen nanofiber assembly is associated with Na^+^ retention [19, 20]. To assess whether this depends on the counter‐anion, EDS analysis was used to measure the elemental composition of 2.5 mg/mL fibrinogen dried with 150 mM sodium phosphate and 150 mM sodium chloride, respectively (see Table 1). Fibrinogen precipitates dried with 1× PBS and 2.5× PBS served as references. All samples contained C, N, O, and S, consistent with the composition of fibrinogen. Nitrogen and sulfur levels remained constant, confirming comparable protein content independent of the salt condition. Clear differences emerged for salt‐specific elements (Na, K, Cl, P). Fibrinogen assembled with 150 mM sodium phosphate showed substantial sodium and phosphorus incorporation accompanied by elevated oxygen content, demonstrating strong phosphate retention. These interactions correlate with the more defined fiber morphology observed by SEM.

**TABLE 1:** Elemental composition of fibrinogen‐salt precipitates on plasma‐cleaned glass slides obtained by SEM‐EDS analysis in low‐vacuum mode. All samples were prepared from 2.5 mg/mL fibrinogen solutions with the different salts, crosslinked with FA vapor, and later rinsed with water, dried, and not sputter‐coated. All values are reported in atomic percent (at%) for nitrogen [N], and the values for all other elements are expressed as ratios normalized to [N]. The normalized ratios represent the average of five different positions per sample.

Salt condition	Morphology		Normalized ratio						
		[N]	[C]/[N]	[O]/[N]	[S]/[N]	[Na]/[N]	[P]/[N]	[K]/[N]	[Cl]/[N]
150 mM NaCl	Macroporous precipitates	19.0	2.34	1.85	0.04	0.03	—	—	0.01
150 mM Na‐PO_4_	Highly porous and well‐formed nanofibers	18.8	2.26	2.05	0.02	0.22	0.06	—	—
1x PBS	Macroporous network with nanofibrous features	18.6	2.26	1.80	0.02	0.16	0.01	0.07	0
2.5x PBS	Macroporous network with nanofibrous features	18.9	2.13	1.89	0.03	0.18	0.02	0.08	0.01

In contrast, fibrinogen dried with 150 mM NaCl contained only trace amounts of sodium and chlorine, indicating weak salt association and efficient removal during washing. This agrees with previous XPS data [20] and confirms that Na^+^ retention is itself anion‐dependent: Na^+^ is co‐retained specifically in the presence of phosphate, consistent with Na^+^‐phosphate–protein ion‐pair formation, indicating that phosphate anions play the dominant role in driving ordered fiber assembly, while cations modulate the resulting network architecture. Similar to SEM analysis, fibrinogen samples prepared with 1× and 2.5× PBS showed intermediate elemental profiles, retaining Na, K, P, and Cl at comparable levels. Unlike previous XPS measurements, EDS also detected potassium and chlorine, likely due to its greater penetration depth [36, 37].

### 3.3 Turbidity as an Indicator of Nanofiber Assembly

To understand whether the differences in morphology and elemental composition of dried fibrinogen precipitates originate from protein–salt interactions in solution, turbidity measurements were performed using UV/vis spectroscopy. This method is well established to study fiber formation of proteins like collagen or fibrin in situ [38, 39, 40, 41]. Unlike Aq roughness analysis, which reflects surface effects during drying [19, 20], turbidity monitors bulk aggregation kinetics [26, 42, 43, 44] and allows long‐term observation of fibrinogen–salt interactions, which is important during fibrillogenesis in thrombin‐free buffer systems.

**FIGURE 3:**
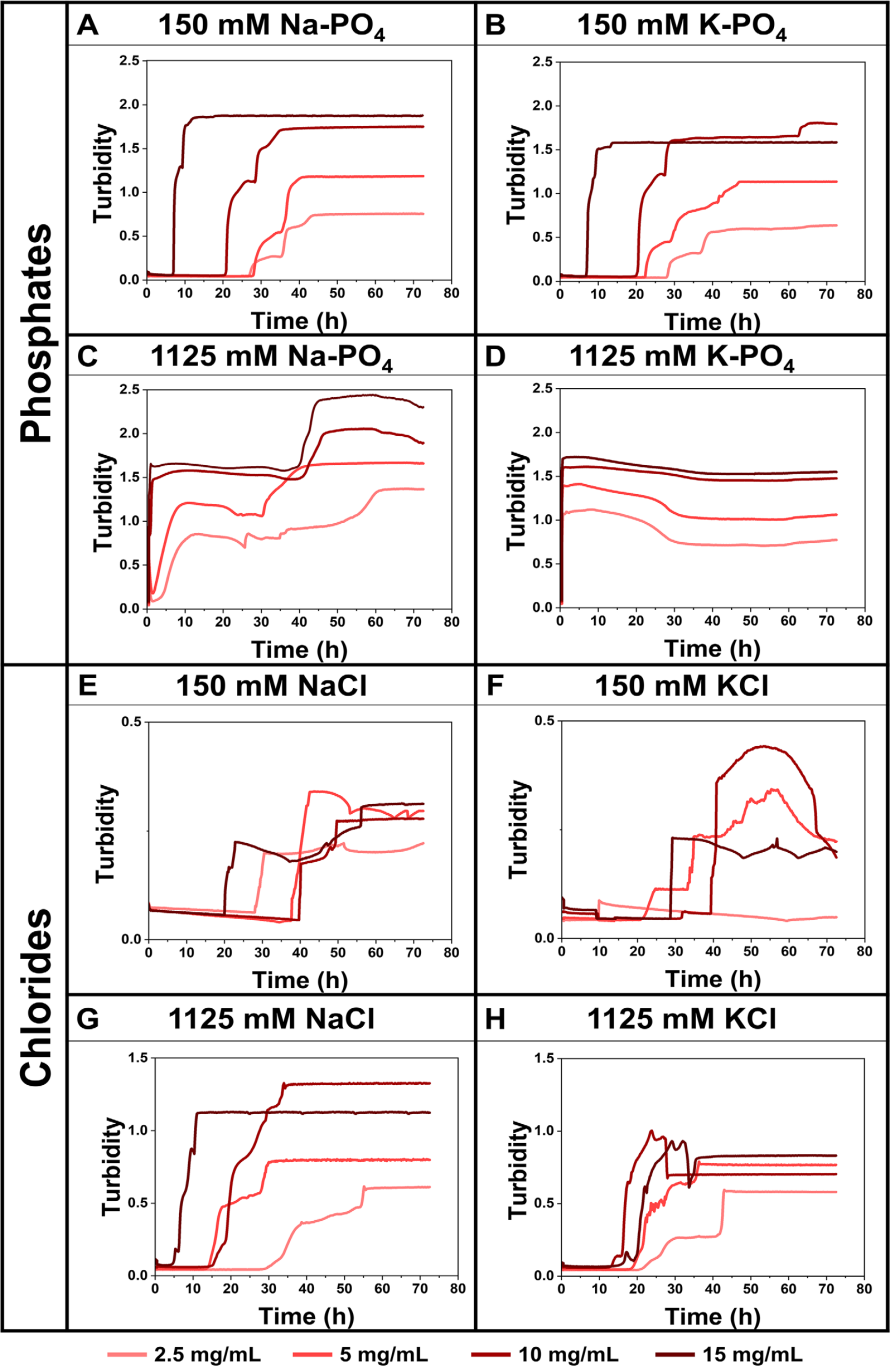
In situ monitoring of turbidity changes during the drying of fibrinogen (Fg) at different concentrations in the presence of different salts. Time‐dependent turbidity profiles were obtained from UV/vis‐measurements at 330 nm. Each graph represents different Fg concentrations in 5 mM Tris with (A) 150 mM Na‐PO_4_, (B) 150 mM K‐PO_4_, (C) 1125 mM Na‐PO_4_, (D) 1125 mM K‐PO_4_ (red), (E) 150 mM NaCl, (F) 150 mM KCl, (G) 1125 mM NaCl, and (H) 1125 mM KCl.

With 150 mM sodium or potassium phosphate, fibrinogen solutions exhibited characteristic three‐phase sigmoidal turbidity curves (Figure 3A,B), clearly distinct from Tris‐only or salt‐only controls (c.f Figure S5A,B). Increasing fibrinogen concentration from 2.5 to 15 mg/mL increased the maximum turbidity, consistent with the known correlation between absorbance and protein concentration [45]. The initial lag phase, shorter at higher protein concentrations, likely reflects formation of early oligomers and nanofibrils, as known from collagen and fibrin assembly [40, 44, 46, 47, 48, 49]. The turbidity profiles of 2.5 mg/mL fibrinogen in 150 mM sodium and potassium phosphate resembled previously reported Aq roughness curves, showing two initial peaks followed by a steep third rise [20]. For all fibrinogen concentrations in 150 mM phosphate salts, the subsequent growth phase showed a rapid turbidity increase whose slope increased with protein concentration. A final plateau indicated complete drying and was higher for sodium than potassium phosphate, suggesting more defined nanofibers consistent with our SEM analysis. Overall, increasing fibrinogen concentration led to earlier onset, faster growth, and higher final turbidity, reflecting accelerated fibrillation and increased nanofiber density.

In contrast, 1125 mM sodium or potassium phosphate showed no lag phase (Figure 3C,D) and did not yield nanofibers upon drying, indicating suppression of nucleation (Figure 1). The turbidity profiles exhibited a two‐step pattern characterized by immediate high turbidity without a lag phase, followed by fluctuations for Na^+^ or gradual decreases for K^+^. These profiles indicate rapid, unstable fibrinogen aggregation, consistent with the formation of rough, porous precipitates rather than ordered nanofibers.

In contrast, 150 mM NaCl and KCl produced irregular, low‐intensity turbidity profiles without sigmoidal transitions, largely independent of fibrinogen concentration, indicating the absence of organized fibrillation (see Figure 3E,F). These features are consistent with rapid, disordered aggregation and the nonfibrillar precipitates observed by SEM. At 1125 mM NaCl (Figure 3G), partially sigmoidal, concentration‐dependent curves appeared, but final absorbance values remained lower than with phosphate salts, suggesting limited or incomplete fiber formation. In contrast, 1125 mM KCl yielded predominantly irregular, low‐level turbidity (Figure 3H), consistent with rough, molten aggregates rather than ordered fibers.

Turbidity profiles in 2.5× and 7.5× PBS showed combined phosphate‐ and chloride‐driven effects, with gradual aggregation characteristic of chloride conditions combined with sharper increases typical of phosphate‐mediated fiber assembly (see Figure S6A,B). Overall, turbidity profiles correlated closely with the final morphologies observed by SEM. Sigmoidal curves indicate nucleation‐driven, ordered nanofiber assembly (150 mM phosphate), whereas irregular or low‐intensity profiles reflect rapid, disordered aggregation leading to amorphous precipitates (chloride or excessive salt).

### 3.4 Changes in Salt Concentration and Turbidity During Drying

To track structural evolution during drying, mass loss and turbidity were measured simultaneously for 2.5 mg/mL fibrinogen in 150 mM sodium phosphate or sodium chloride. This concentration was selected because it exhibited the slowest turbidity increase, providing optimal temporal resolution of the aggregation behavior.

**FIGURE 4:**
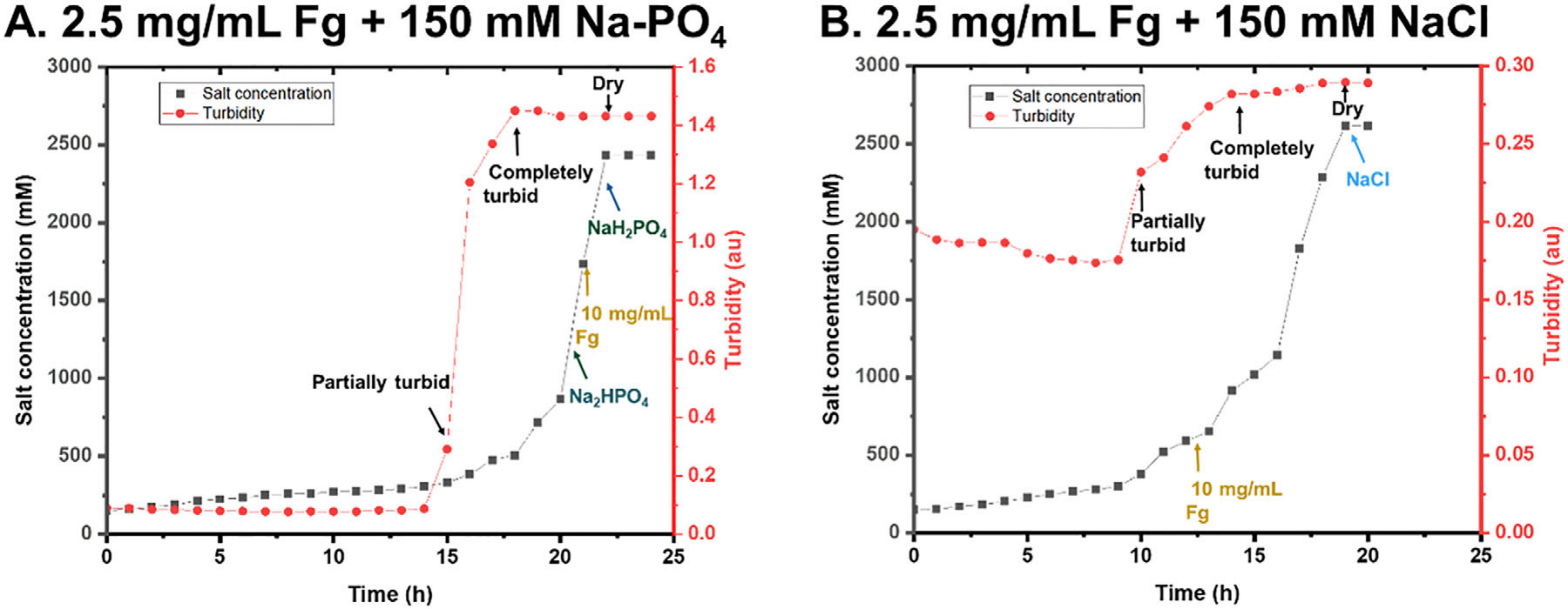
Time‐dependent changes in turbidity and salt concentration during drying of 2.5 mg/mL fibrinogen with (A) 150 mM sodium phosphate and (B) 150 mM NaCl. The combined diagram displays the relationship between turbidity (red lines, right *y*‐axes) and salt concentration (black lines, left y‐axes) over time in a drying fibrinogen (Fg) solution. Mass loss and turbidity data were collected over 24 h to follow how fibrinogen and salt concentration increased during evaporation. The different arrows mark the time point when the respective salt and fibrinogen are expected to reach their solubility limits and precipitate. Overall, fibrinogen assembled more slowly in chloride conditions compared to phosphate conditions.

For sodium phosphate, periodic weighing altered the drying profile: only two turbidity peaks were observed instead of the three seen under undisturbed conditions, likely due to disruption of concentration gradients and accelerated evaporation (Figure 4). Before the main growth phase, phosphate and fibrinogen concentrations reached 308 mM and 5.2 mg/mL, respectively. Turbidity began to rise after approximately 15 h (about 62% of total drying time), marking the onset of visible aggregation and fibril formation. Between 15 and 18 h, turbidity increased sharply, accompanied by substantial concentration increases, and plateaued upon completion of drying. These stages correspond to progressive protein aggregation, network formation, and densification, consistent with nucleation‐driven nanofiber assembly observed under phosphate conditions.

In contrast, sodium chloride produced much lower turbidity throughout drying. During the first 10 h, turbidity remained nearly constant despite increasing salt concentration, followed by only modest increases that reached a plateau upon complete drying.

Time‐lapse imaging revealed distinct precipitation patterns for the two salts. Sodium phosphate produced largely homogeneous, progressively opaque layers across the droplet surface during drying, indicating bulk precipitation of fibrinogen and sodium phosphate throughout the film (see Figure S7). In contrast, NaCl crystallization occurred only at later stages and initiated at the droplet edges, forming faceted crystals that propagated inward (see Figure S8).

The combined mass loss–turbidity data reveal distinct precipitation pathways for phosphate and chloride systems. In phosphate solutions, disodium hydrogen phosphate is expected to precipitate first upon supersaturation [50], temporarily reducing ionic strength, followed by fibrinogen and finally monosodium dihydrogen phosphate. This sequential precipitation likely underlies the multi‐step turbidity behavior. In contrast, in sodium chloride solutions, fibrinogen is expected to precipitate before NaCl due to strong salting‐out effects, while salt crystallization occurs only at later stages. This simpler sequence explains the lower, less structured turbidity profiles observed with chloride compared to phosphate systems.

### 3.5 Effect of Ionic Environment on the Hydrodynamic Radius of Fibrinogen

Fibrinogen is a flexible, elongated plasma glycoprotein whose conformation depends strongly on electrolyte composition, ionic strength, and pH [51, 52, 53, 54, 55, 56]. To clarify whether the sigmoidal turbidity profiles observed at 1125 mM NaCl reflect true nucleation‐driven assembly or random clustering, early aggregation was studied in solution using time‐resolved DLS to determine the hydrodynamic radius (R_h_).

Using 0.5 mg/mL fibrinogen and low sodium phosphate concentrations between 50 and 150 mM, the initial R_h_ was 13.5 ± 0.5 nm (see Table 2), consistent with the radius of native monomeric fibrinogen molecules in ionic solution [25, 54]. Over 3 h, R_h_ increased progressively and concentration‐dependently up to 154.5 ± 20.7 nm at 150 mM, indicating slow self‐association under physiological phosphate conditions. However, at 300 mM phosphate, R_h_ was already elevated at t = 0 h (R_h_ = 26.72 ± 3.40 nm) and further increased with time, suggesting immediate and ongoing fibrinogen aggregation. Above 600 mM phosphate, rapid aggregation instantly produced large, heterogeneous clusters that prevented reliable R_h_ analysis. These findings agree with SEM data of fibrinogen in these phosphate conditions, which show suppression of nanofiber formation at high concentrations (see Figure S9), indicating that phosphate‐rich environments promote rapid, uncontrolled aggregation over ordered fibrillogenesis.

**TABLE 2:** Hydrodynamic radius (R_h_) of 0.5 mg/mL fibrinogen measured by DLS in the presence of varying concentrations of sodium phosphate (Na‐PO_4_) and sodium chloride (NaCl) at t = 0 h and after 3 h of incubation. Values represent the mean ± standard deviation.

	Na‐PO_4_			NaCl		
Salt concentration	R_h_ = 0 h	R_h_ = 3 h	Morphology	R_h_ = 0 h	R_h_ = 3 h	Morphology
50 mM	13.09 ± 0.13 nm	42.22 ± 1.56 nm	Fibers	13.20 ± 0.10 nm	15.29 ± 0.41 nm	No fibers
100 mM	13.01 ± 0.21 nm	99.14 ± 1.80 nm	Fibers	13.88 ± 0.26 nm	18.88 ± 1.35 nm	No fibers
150 mM	13.45 ± 0.48 nm	154.57 ± 20.71 nm	Fibers	14.30 ± 0.13 nm	26.30 ± 0.86 nm	No fibers
300 mM	26.72 ± 3.40 nm	237.1 ± 16.61 nm	Fibers	14.96 ± 0.13 nm	34.89 ± 3.22 nm	No fibers
600 mM	1561 ± 168.8 nm*	2137 ± 218.8 nm*	No fibers	15.70 ± 0.22 nm	49.11 ± 1.52 nm	No fibers
1000 mM	3328 ± 189.7 nm*	3422 ± 153.2 nm*	No fibers	16.36 ± 0.12 nm	135.8 ± 17.11 nm	No fibers

***Note***: Asterisks indicate that R_h_ values at ≥ 600 mM Na‐PO_4_ are not quantitative due to rapid, heterogeneous aggregation causing multi‐mode correlation decays. Morphological features (fibers vs. no fibers) were determined from complementary SEM analysis after the samples were dried and cross‐linked.

In contrast, NaCl induced only minor changes in R_h_ between 50 and 600 mM, with fibrinogen remaining predominantly monomeric or forming small oligomers. A pronounced increase in R_h_ was observed only at 1000 mM NaCl after 3 h, indicating limited aggregation at very high ionic strength. This aligns with the corresponding SEM data, which show no fiber formation in these chloride systems (see Figure S9).

Overall, the strong R_h_ increase with phosphate cannot be explained by simple charge screening, which typically reduces protein‐protein interactions [57]. Instead, a critical range between 150 and 300 mM sodium phosphate appears to shift fibrinogen in solution from stable monomers toward aggregation.

### 3.6 Intermediate Structures During Nanofiber Formation

Recent studies by Saha et al. and Hämisch et al. demonstrated that fibrinogen fiber assembly follows a two‐step self‐assembly mechanism triggered by reduced ionic strength in mixed sodium chloride/sodium phosphate systems [23, 25]. To investigate the sequential events during fibrinogen precipitation with these anions, we monitored the drying of 2.5 mg/mL fibrinogen in 150 mM sodium phosphate or sodium chloride using environmental SEM. Drying was interrupted at defined time points by removing excess liquid. Samples were first analyzed in low vacuum without cross‐linking or coating to preserve native‐like morphology [58] and detect salt crystallization, then cross‐linked with formaldehyde vapor, washed, and gold‐coated for subsequent high‐resolution SEM.

**FIGURE 5:**
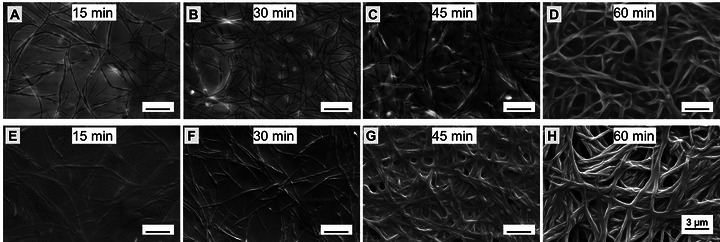
Time‐series SEM analysis of nanofiber assembly with 2.5 mg/mL fibrinogen and 150 mM sodium phosphate. Samples were first analyzed on a plasma‐cleaned glass in low‐vacuum mode at 70 Pa without any cross‐linking or gold coating using 5 kV after assembly times of (A) 15, (B) 30, (C) 45, and (D) 60 min. With increasing time, the emergence of individual nanofibrils is clearly visible until a dense nanofiber network is formed. Subsequently, the same fiber networks were cross‐linked with FA vapor, sputter‐coated with gold, and analyzed in high vacuum mode at 5 kV for assembly times of (E) 15, (F) 30, (G) 45, and (H) 60 min. The different phases of fiber assembly and increasing compaction of the fiber network confirm the trends identified in low‐vacuum analysis.

This in situ analysis in low vacuum revealed progressive nanofiber formation in sodium phosphate (Figure 5A–D), confirmed by high‐vacuum SEM (Figure 5E–H). After 15 min, sparse elongated nanofibers appeared, indicating early nanofiber formation. At 30 min, fibril density and interfibrillar junctions increased, consistent with elongation and lateral association observed in turbidity measurements (c.f. Figure 3). By 45 min, an interconnected network formed through fiber bundling, producing thicker fibers and smaller pores. After 60 min, a dense, interconnected nanofibrous network with well‐defined pores formed, matching the morphology of fully dried samples. No sodium phosphate crystals were detected during drying, consistent with the macroscopically uniform appearance of the mixture (Figure S7). The observed time‐ and concentration‐dependent evolution suggests a nucleation‐elongation mechanism for phosphate‐driven fibrillation, similar to other self‐assembling polymer systems such as trioxane or oligo(m‐phenylene ethynylene) imines [59].

**FIGURE 6:**
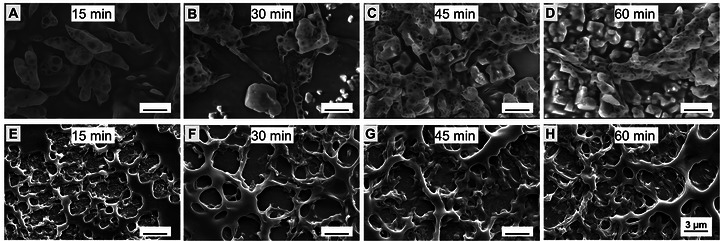
Time‐series SEM analysis of precipitates formed through drying 2.5 mg/mL fibrinogen with 150 mM NaCl. Samples were first analyzed in low‐vacuum mode at 70 Pa without any cross‐linking or gold coating using 5 kV after assembly times of (A) 15, (B) 30, (C) 45, and (D) 60 min. With increasing time, the emergence of fibrinogen aggregates is clearly visible until clusters of fibrinogen aggregates and salt crystals are formed. Subsequently, the fibrinogen aggregates were cross‐linked with FA vapor, sputter‐coated with gold, and analyzed in high vacuum mode at 5 kV for assembly times of (E) 15, (F) 30, (G) 45, and (H) 60 min. For all time points, molten aggregates were found that did not contain any crystals anymore.

In contrast, drying 2.5 mg/mL fibrinogen with 150 mM NaCl produced no nanofibers. Environmental SEM (Figure 6A–D) and high‐vacuum SEM (Figure 6E–H) revealed distinct, non‐fibrous morphologies. After 15 min, rough, crystal‐like features appeared in low vacuum, while high‐vacuum images revealed molten, irregular aggregates. From 30 to 60 min, crystal and aggregate numbers increased in low vacuum, whereas aggregate morphology remained largely unchanged in high vacuum. The final structure matched the irregular, macroporous morphology of completely dried samples (Figure S8), consistent with nonspecific aggregation driven by chloride, which primarily screens electrostatic interactions without strongly structuring water. This pathway resembles step‐growth aggregation as seen in our turbidity analysis and is known to yield amorphous, macroporous clusters rather than organized nanofibers [60, 61].

Morphological differences between low‐ and high‐vacuum SEM images of chloride‐containing samples likely result from residual water in non‐cross‐linked samples. Dark, indented regions in low‐vacuum images (Figure 6A–D) suggest incomplete drying, explaining deviations from cross‐linked samples. Residual water was observed in NaCl‐containing samples but not in sodium phosphate systems. Although phosphate ions have stronger hydration shells and are expected to retain more water, the opposite behavior likely reflects microstructural effects: NaCl–fibrinogen blends form compact crystalline structures that trap water, whereas porous fibrillar networks formed with sodium phosphate permit more efficient water release [62, 63, 64].

Consistent with this interpretation, macroscopic analysis showed dendritic NaCl crystals during drying (Figure S8), but no residual crystals were detected after cross‐linking, washing, and drying (cf. Figures 6E–H and 2A–D). EDS analysis showed negligible Na and Cl signals in these samples (Table 1), indicating that crystalline NaCl is removed during processing. In contrast, sodium detected in phosphate‐assembled fibrinogen fibers is therefore likely protein‐bound rather than present as residual salt. Together, these findings support a model in which transient NaCl crystallites promote compact, water‐retaining aggregates that dissolve upon washing, whereas phosphate drives the formation of open fibrillar networks that release water efficiently and retain specifically associated ions.

Our results indicate that multivalent, kosmotropic phosphate anions modulate fibrinogen primarily through specific ion–protein interactions rather than bulk ionic strength effects. This behavior is consistent with the broader Hofmeister series pairing established in Stamboroski et al., indicating that the phosphate–chloride contrast reflects a general principle of anion‐directed fibrinogen fibrillogenesis rather than a salt‐specific effect limited to a particular ion pair [20]. Molecular dynamics simulations have identified substantial structural variability of fibrinogen in solution [51] and revealed phosphate binding within the inner Helmholtz layer [20], supporting the concept of polyvalent anion‐mediated ion bridging. These simulations further show preferential phosphate accumulation and significantly longer residence times compared to chloride, supporting specific anion binding rather than purely electrostatic screening [20, 65]. Owing to its high charge density and hydrogen‐bonding capacity, sodium phosphate likely enhances these interactions, promoting intermolecular association.

Phosphate anions can establish multiple electrostatic and hydrogen‐bonding interactions with positively charged and polar residues, potentially bridging fibrinogen domains or adjacent molecules [66, 67]. Such interactions may expand the fibrinogen molecule and expose binding sites, particularly within the coiled‐coil regions and flexible αC domains, which are proposed as likely regions for anion coordination [51, 68, 69]. Charge screening at moderate phosphate levels likely reduces intramolecular repulsion and, together with dehydration and partial charge neutralization, may facilitate early‐stage clustering and nucleation [68], followed by directional end‐to‐end association into protofibrils [66, 67, 69, 70, 71, 72], that laterally assemble into nanofibers and ultimately a porous network [53, 73, 74, 75]. This suggested mechanism is supported by our previous observation of partial α‐helix to β‐strand transitions during fiber assembly of fibrinogen [14]. FTIR data from our previous study demonstrate phosphate‐specific amide I band shifts consistent with a partial α‐helix to β‐strand transition, which is absent in chloride conditions [20]. The kosmotropic character of phosphate anions reduces water activity and strengthens protein hydration, while drying removes water and stabilizes the resulting hierarchical architecture [20, 76]. Unlike fibrin, these salt‐induced fibrinogen fibers remain non‐crosslinked and water‐soluble [13]. At high ionic strength, however, excessive charge screening promotes rapid, disordered aggregation rather than controlled fiber assembly, as also reported for other filamentous proteins and nanoparticles [77, 78]. This concentration‐dependent crossover from specific anion–protein interaction at moderate phosphate concentrations to non‐specific Debye screening at extreme concentrations explains the non‐monotonic dependence of fibrinogen assembly on phosphate concentration and is consistent with the suppression of sigmoidal kinetics in turbidity above 600 mM. At these high ionic strengths, electrostatic interactions are strongly screened, such that apparent ion retention reflects non‐specific co‐precipitation or salt entrapment rather than specific adsorption to fibrinogen, thereby preventing the formation of ordered nanofiber structures.

In contrast, chloride ions inhibit fibrinogen fiber formation primarily through nonspecific electrostatic screening rather than structural mediation [20]. As a monovalent, weakly coordinating ion, chloride reduces long‐range fibrinogen–fibrinogen interactions without promoting intermolecular bridging or structural rearrangement. Consistent with the Hofmeister series, chloride does not substantially disturb the protein hydration shell or native structure [76], and therefore fails to induce the directional contacts required for nanofiber assembly.

Cation‐specific effects further support this mechanism: sodium phosphate produced more porous fiber networks than potassium phosphate, and sodium was retained in phosphate‐assembled fibers but not in chloride systems in both the present study and our previous work [20], indicating strong phosphate–protein association and ion‐pair stabilization. Phosphate forms stable ion pairs with Na^+^ cations and binds to protein surfaces, whereas chloride interacts weakly and does not retain sodium upon drying. The porous fiber networks observed with in situ SEM analysis during drying are consistent with diffusion‐limited cluster aggregation, producing fractal‐like networks rather than compact aggregates and suggesting a hierarchical supramolecular polymerization pathway analogous to chain‐growth polymerization processes [60]. Previous studies have characterized the surface structure and hydration‐dependent roughness of fibrinogen nanofiber scaffolds using AFM, as well as their mechanical behavior by bulk testing, demonstrating the formation of stable, hydrated fiber networks [15, 16]. In line with these scaffold characteristics, the biological compatibility of fibrinogen nanofiber scaffolds, including platelet interaction, fibroblast–keratinocyte co‐culture, and hematopoietic stem cell interaction, has been demonstrated in previous studies [16, 17, 18, 79].

To assess the robustness of the nucleation‐elongation mechanism across different drying kinetics, fibrinogen assembly was additionally examined at 10% and 50% relative humidity using 2.5 mg/mL fibrinogen in 150 mM sodium phosphate (see Figure S10). Fibrous networks formed under all tested humidity conditions, indicating that the assembly mechanism is robust across the investigated range of drying conditions.

Overall, multivalent phosphate anions act as tunable drivers of fibrinogen self‐assembly, promoting controlled fibrillogenesis within a defined salt concentration window, and disordered aggregation at higher ionic strength, whereas monovalent chloride anions primarily exert nonspecific ionic strength effects. These findings highlight how ion valency and hydration properties govern protein‐protein interactions and supramolecular fibrinogen organization during the transition from the liquid phase to the solid state.

Beyond the mechanistic insights reported here, the enzyme‐free, phosphate‐driven assembly route offers practical advantages for scaffold fabrication. Unlike thrombin‐induced fibrin polymerization, this approach avoids the use of clotting enzymes, as well as fibrin degradation products, and residual thrombin activity. This establishes a framework for the development of fibrinogen‐based biomaterials with tunable architectures.

## 4 Conclusion

In this study, we show that phosphate ions play a decisive role in directing the self‐assembly of fibrinogen into nanofibrillar networks in the absence of thrombin. In contrast, chloride yields amorphous aggregates rather than ordered fibers. Light‐scattering and environmental/high‐vacuum SEM analyses indicate a nucleation‐elongation mechanism in which small aggregates evolve into interconnected nanofibers with increasing fibrinogen and phosphate concentrations. As a kosmotropic anion, phosphate likely reduces intermolecular electrostatic repulsion and promotes specific ion‐protein interactions that expose interaction sites, including the flexible αC regions and coiled‐coil linkers, which are proposed as likely regions of anion coordination. The resulting nanofibers closely resemble thrombin‐induced fibrin networks but are formed entirely through physicochemical interactions.

Overall, our results identify multivalent kosmotropic anions like phosphate as tunable regulators of fibrinogen fiber assembly, lowering the nucleation barrier and defining distinct morphological regimes across the fibrinogen‐phosphate phase space. Beyond mechanistic insight, this phosphate‐driven pathway provides a facile, enzyme‐independent route to fabricate fibrinogen nanofibers with controllable fiber density and network architecture, offering a valuable platform for bioinspired scaffolds for tissue engineering and regenerative medicine.

## Conflicts of Interest

The authors declare no conflicts of interest.

## Supporting information




**Supporting File**: mabi70203‐sup‐0001‐SuppMat.pdf.

## Data Availability

The data that support the findings of this study are available from the corresponding author upon reasonable request.
