# Supplementary material for: Anion‐Specific Mechanisms in Fibrinogen Self‐Assembly: Contrasting Effects of Phosphates and Chlorides in Nanofiber Formation
Source: Macromol Biosci. 2026 Jun 14;26(6):e70203. doi: 10.1002/mabi.70203 (PMC13265632; doi:10.1002/mabi.70203)
Supplement: Supplementary file 1 — Supporting File: mabi70203‐sup‐0001‐SuppMat.pdf. [file MABI-26-e70203-s001.pdf]

Supplementary Information

Anion-Specific Mechanisms in Fibrinogen Self-assembly: Contrasting Effects of Phosphates and Chlorides in Nanofiber Formation

Antoine Eyram Kwame[1], *Aparna Sai Malisetty*[2], *Michael Maas*[3,4], *Susan Köppen-Hannemann*[2,4], *Lucio Colombi Ciacchi*[2,4], *Dorothea Brüggemann*[1,4]

[1] Biophysics and Applied Biomaterials, Hochschule Bremen - City University of Applied Sciences, Bremen, Germany

[2] Hybrid Materials Interfaces Group, Faculty of Production Engineering and Bremen Center for Computational Materials Science, University of Bremen, Bremen, Germany

[3] Advanced Ceramics, University of Bremen, 28359 Bremen, Germany

[4] MAPEX Center for Materials and Processes, University of Bremen, 28359 Bremen, Germany

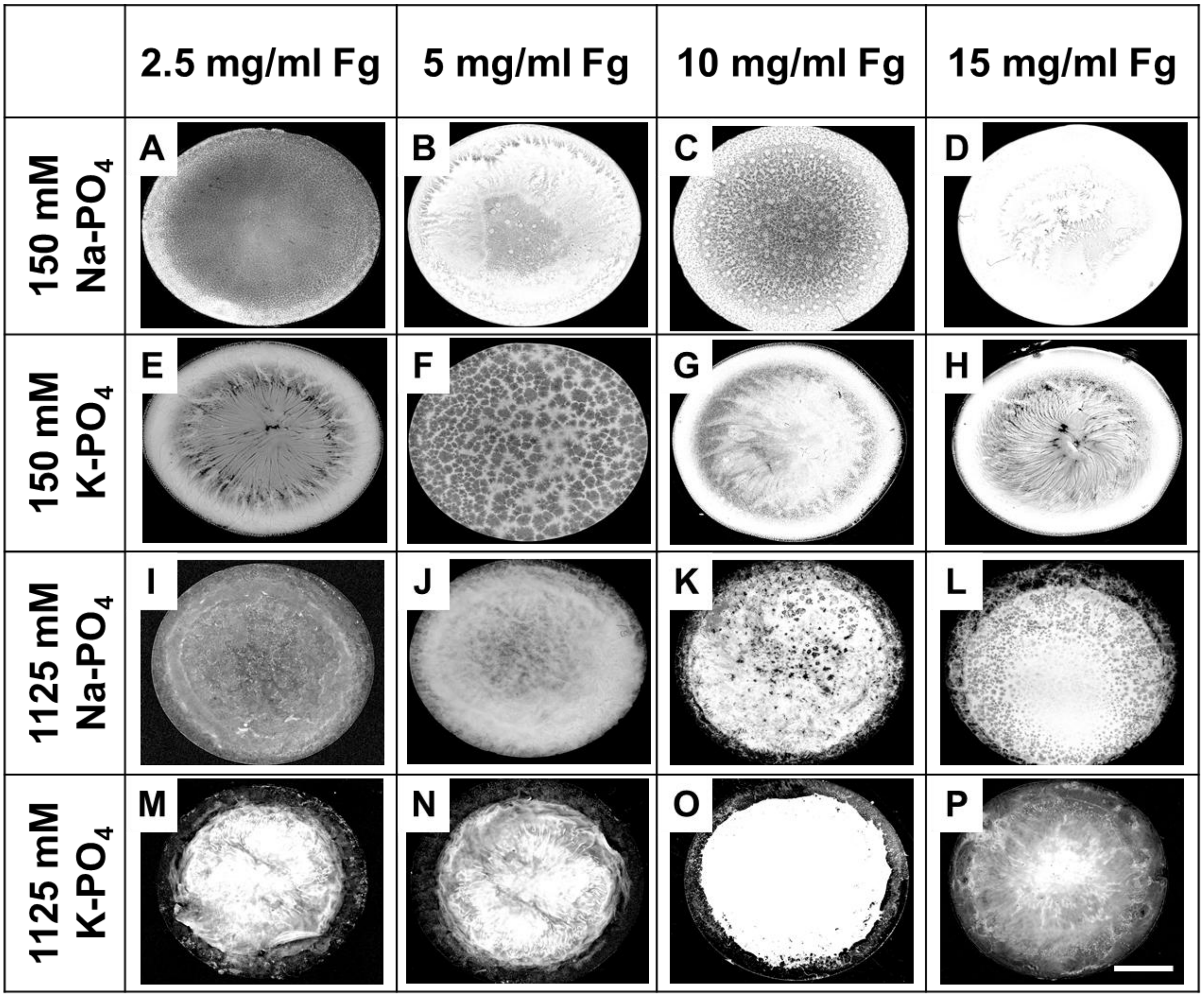

Figure S1: Light microscope images of different concentrations of fibrinogen samples dried in the presence of sodium and potassium phosphate without crosslinking and washing. When fibrinogen from (A) 2.5 mg/ml, (B) 5 mg/ml, (C) 10 mg/ml, and (D) 15 mg/ml samples was dried in the presence of 150 mM sodium phosphate, a smooth surface was observed. Fibrinogen from (E) 2.5 mg/ml to (F) 5 mg/ml, (G) 10 mg/ml and (H) 15 mg/ml with 150 mM potassium phosphate also yielded smooth surfaces. When (I) 2.5 mg/ml to (J) 5 mg/ml, (K) 10 mg/ml and (L) 15 mg/ml fibrinogen were dried with 1125 mM sodium phosphate and (M) 2.5 mg/ml to (N) 5 mg/ml, (O) 10 mg/ml and (P) 15 mg/ml fibrinogen were dried in the presence of 1125 mM potassium phosphate the surface varied from that of the lower salt concentrations, as highly turbid and viscous solutions were immediately formed. Upon drying, the viscous mixture formed a thick film on the glass slides. Scale bar represents 3 cm

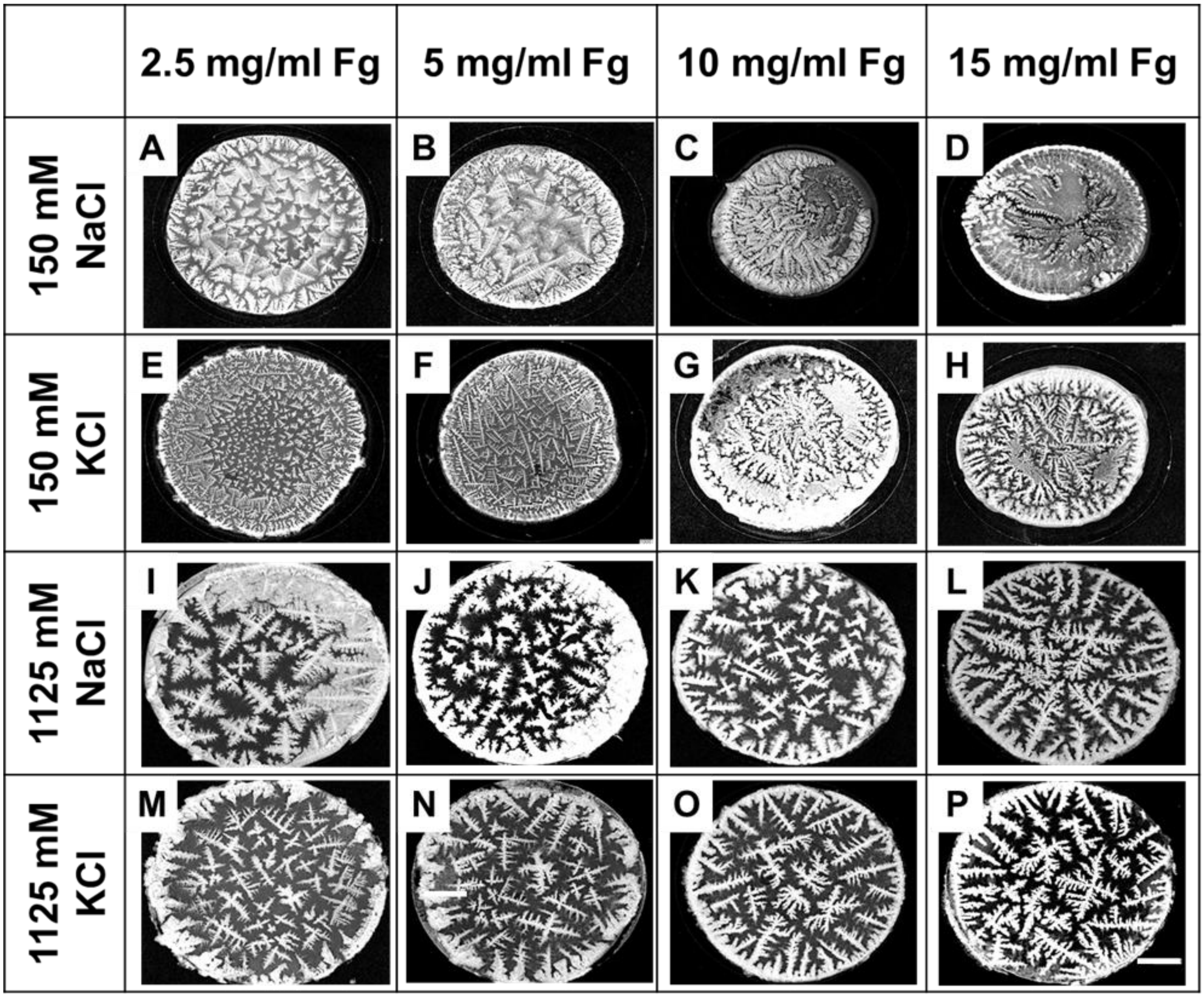

Figure S2: Light microscope images of different concentrations of fibrinogen samples dried in the presence of chloride salts without crosslinking and washing. When fibrinogen from (A) 2.5 mg/ml (B) 5 mg/ml, (C) 10 mg/ml and (D) 15 mg/ml nanofibers was dried in the presence of 150 mM NaCl, a rough surface with dendritic structures was observed. Fibrinogen from (E) 2.5 mg/ml to (F) 5 mg/ml, (G) 10 mg/ml and (H) 15 mg/ml with 150 mM KCl also yielded rough, uneven surface features with dendritic structures. When (I) 2.5 mg/ml to (J) 5 mg/ml, (K) 10 mg/ml and (L) 15 mg/ml fibrinogen were dried with 1125 mM NaCl, and (M) 2.5 mg/ml to (N) 5 mg/ml, (O) 10 mg/ml and (P) 15 mg/ml fibrinogen were dried in the presence of 1125 mM KCl, highly crystalline and dendritic surfaces were found. Overall, the higher chloride concentrations formed bigger salt crystals in the fibrinogen precipitates than the low chloride concentrations. Scale bar represents 3 cm

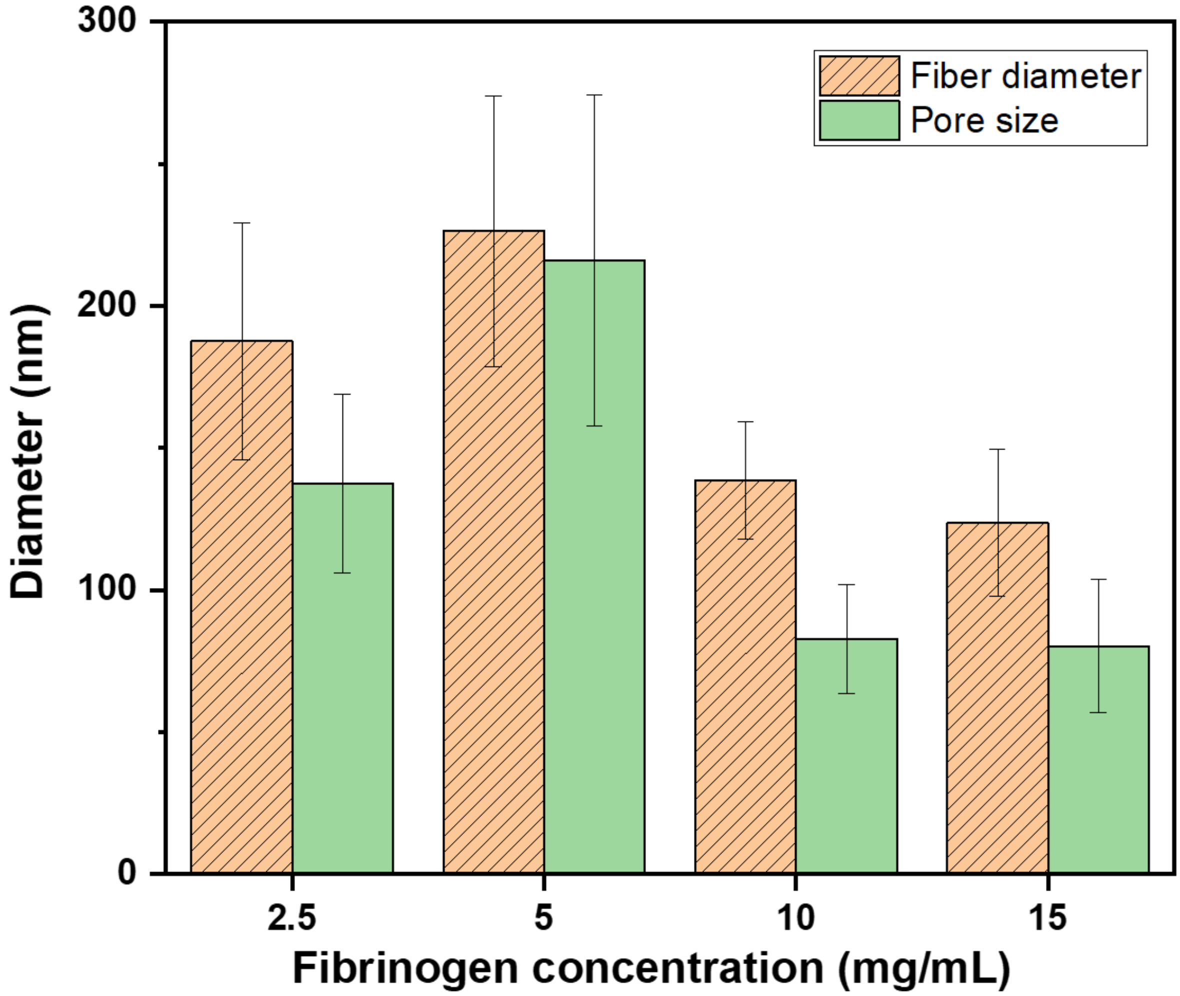

Figure S3: Average fiber and pore diameters of fibrinogen fibers assembled with 150 mM sodium phosphate. The average fiber diameter and average pore size decreased with increasing fibrinogen concentration except for 5 mg/ml. For fibrinogen precipitates with sodium phosphate, 5 mg/mL fibrinogen yielded the highest average pore size, while 15 mg/mL fibrinogen exhibited the lowest fiber diameter and smallest pore size indicating a compaction of the fibrous networks.

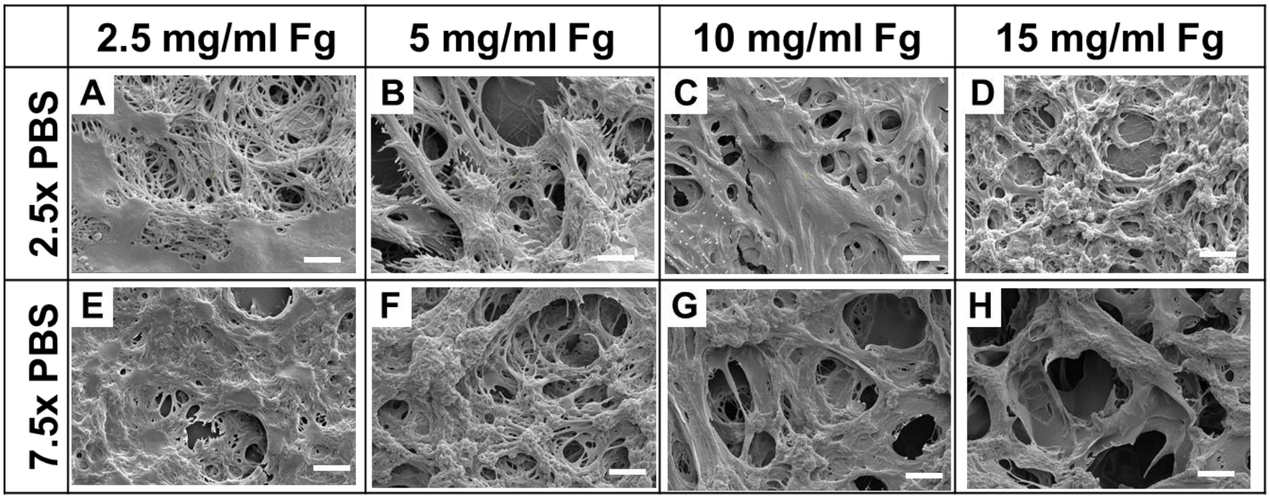

Figure S4: SEM images of fibrinogen at varying concentrations that was dried with PBS (containing a mixture of phosphate- and chloride-salts), crosslinked with FA vapor and subsequently washed. When solutions containing (A) 2.5 mg/mL, (B) 5 mg/mL, (C) 10 mg/mL, and (D) 15 mg/mL fibrinogen were dried 2.5x PBS, fibrous features were found that were overlaid with a nodular structure. When solutions with (E) 2.5 mg/mL, (F) 5 mg/mL, (G) 10 mg/mL, and (H) 15 mg/mL fibrinogen were dried with 7.5x PB only few fibrous features were found for the lower concentrations while with 10 and 15 mg/mL the topography resembled that of fibrinogen precipitates that were formed exclusively with chlorides. Scale bars represent 5 µm.

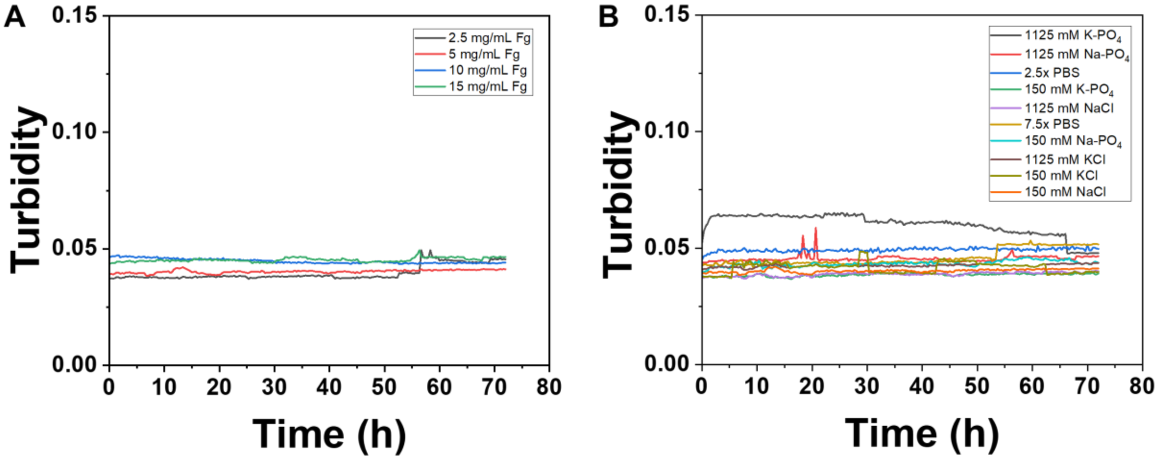

Figure S5. Turbidity measurements of fibrinogen solutions under varying conditions over 72 hours. (A) Turbidity profiles of fibrinogen (Fg) at different concentrations (2.5, 5, 10, and 15 mg/mL) in 5 mM Tris buffer (pH 7.4). All samples exhibit low and stable turbidity values throughout the entire drying period, indicating that no significant self-assembly or aggregation occurs under these conditions. (B) Turbidity of the reference salt solutions: NaCl, KCl, PBS (2.5× and 7.5×), and phosphate salts (Na-PO_4 and K-PO_4 at 150 mM and 1125 mM). Similar to (A), turbidity remains minimal and stable across all salt conditions.

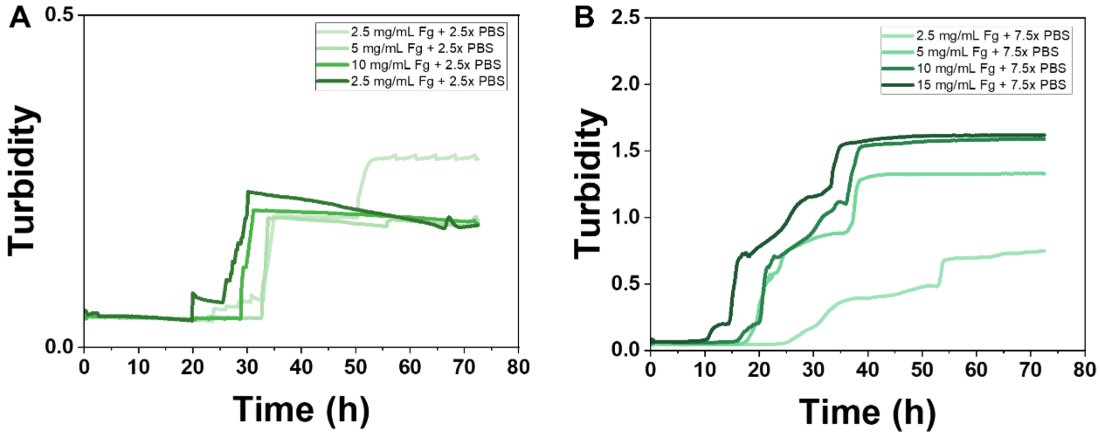

Figure S6: In situ monitoring of turbidity changes during the drying of fibrinogen in the presence of PBS. Time-dependent turbidity profiles were obtained from UV/Vis measurement of fibrinogen (Fg) drying in the presence of (A) 2.5x and (B) 7.5x PBS at 330 nm.

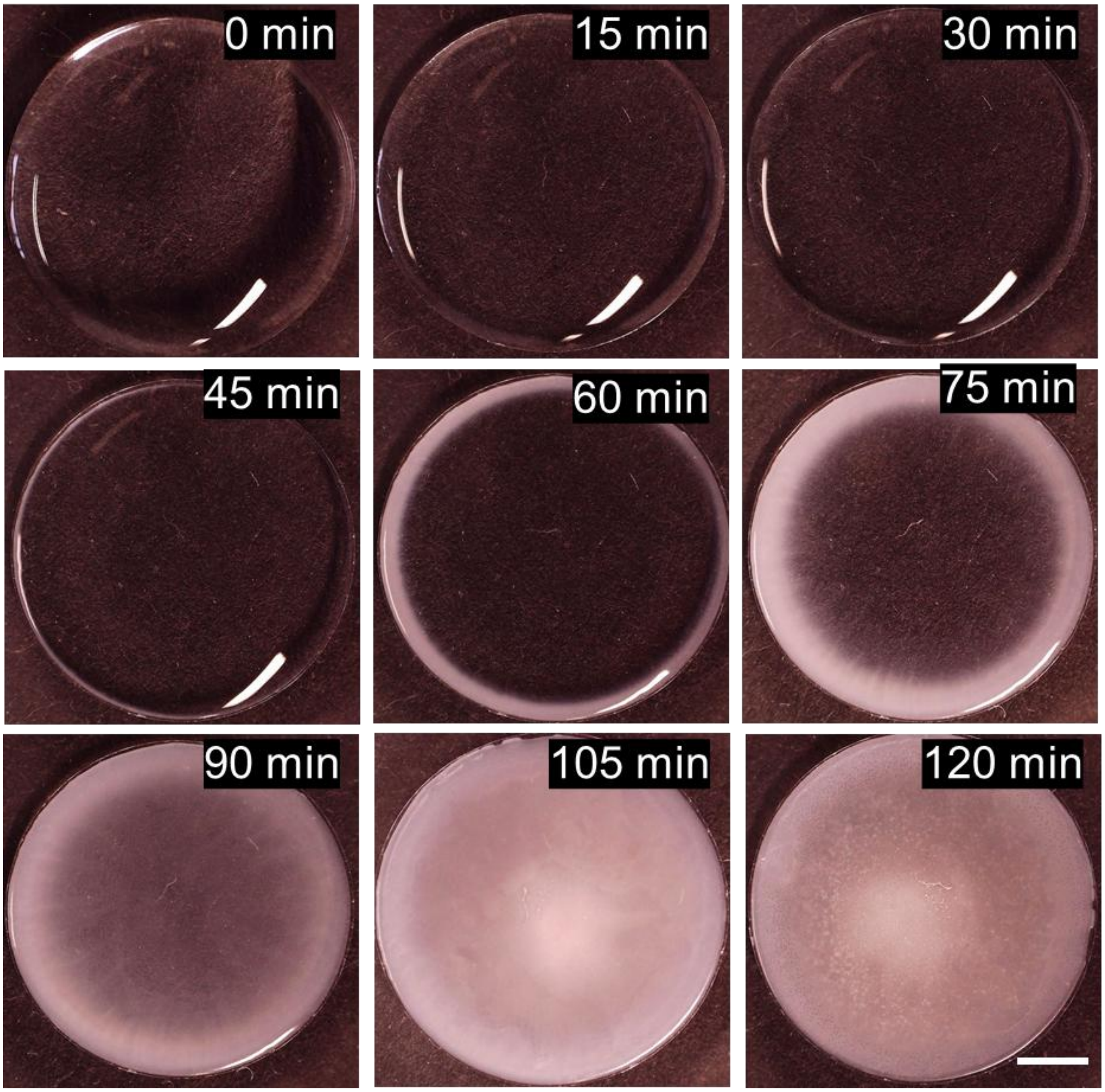

Figure S7*:* Time-lapse microscopy images showing the progressive drying of a 2.5 mg/mL fibrinogen solution in the presence of 150 mM sodium phosphate over 120 minutes. The solution was dried on a 15 mm plasma-cleaned glass. During the initial 45 minutes the solution was transparent. After 60 minutes the droplet gradually became turbid at the outer edges. By 120 minutes, a dense film with uniform turbidity was observed, suggesting complete drying. Scale bar: 3 cm.

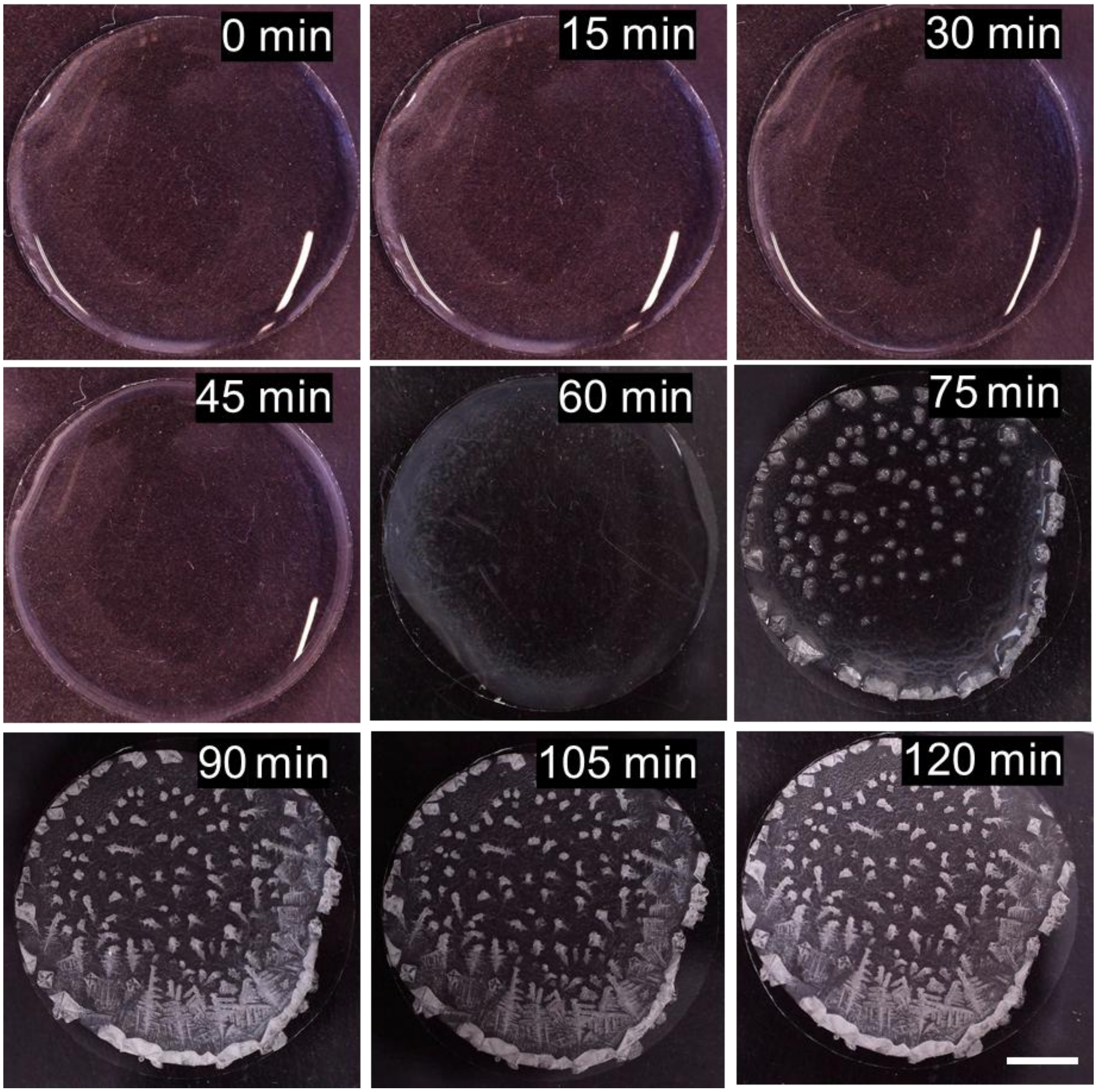

Figure S8: Time-lapse microscopy images showing the drying process of a 2.5 mg/mL fibrinogen solution in the presence of 150 mM NaCl over 120 minutes. The droplet remained largely transparent during the initial drying phase until individual NaCl crystals started to form from the outer edges after 75 minutes. By 90 minutes, NaCl crystals had formed on the whole surface with dendritic structures emerging at the outer edge, and the sample appearance did not change further until 120 minutes. Scale bar: 3 cm

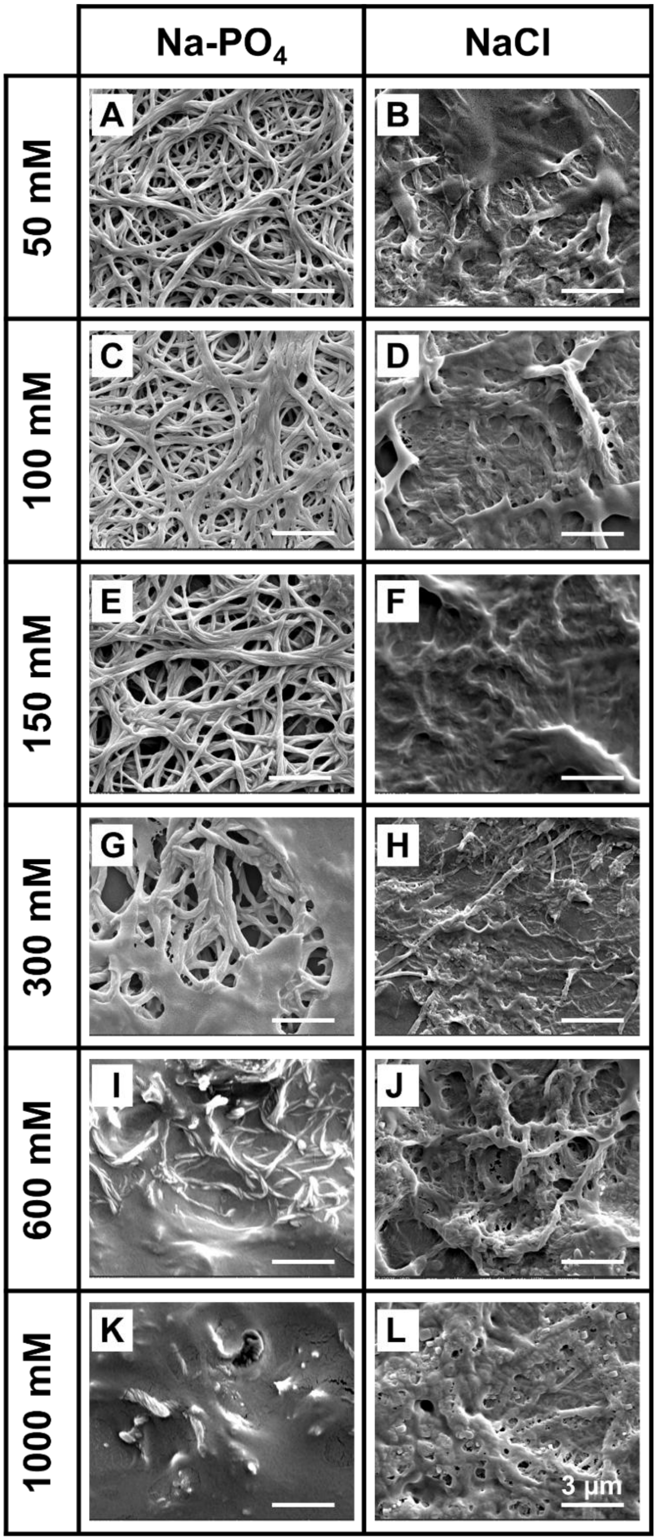

Figure S9: Scanning electron microscopy images of 0.5 mg/mL fibrinogen dried with different concentrations of sodium phosphate (Na-PO_4) and sodium chloride (NaCl). (A, C, E, G) Sodium phosphate at moderate concentrations yields interconnected nanofibrous networks. **(I–K)** Increasing the sodium phosphate concentration to 600 mM and 1000 mM results in non-fibrous aggregates. **(B, D, F, H, J, L)** Chloride-containing samples predominantly form fibrinogen precipitates without any fibrous features across the whole concentration range. Scale bars: 3 µm.

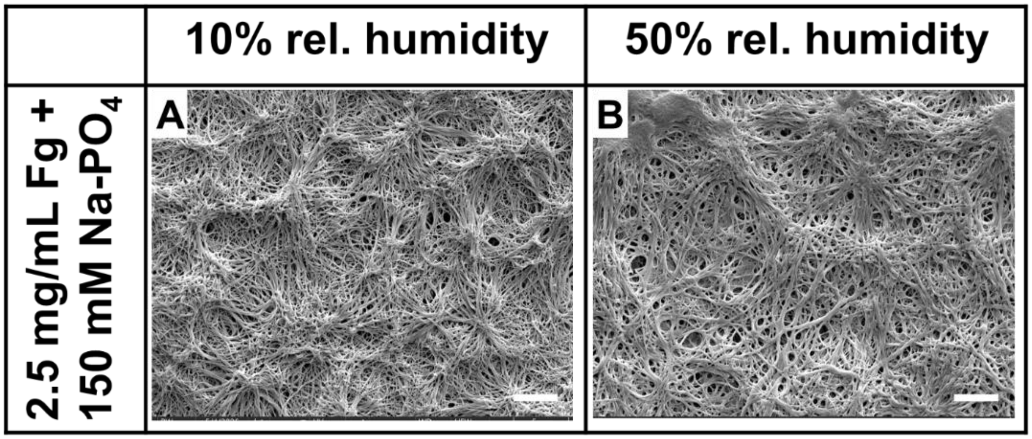

Figure S10: Scanning electron microscopy images of 2.5 mg/mL fibrinogen dried in the presence of 150 mM sodium phosphate (Na-PO_4) at different relative humidities. Samples were crosslinked and washed prior to imaging. (A) At 10% relative humidity, nanofibrous networks are observed. (B) At 50% relative humidity, similar nanofibers were observed. The comparable morphologies indicate that nanofiber assembly is largely independent of humidity under these conditions. Scale bars: 5 µm.
